# The Validity, Reliability, and Sensitivity of a Smartphone-Based Seated Postural Control Assessment in Wheelchair Users: A Pilot Study

**DOI:** 10.3389/fspor.2020.540930

**Published:** 2020-12-17

**Authors:** Mikaela L. Frechette, Libak Abou, Laura A. Rice, Jacob J. Sosnoff

**Affiliations:** ^1^Motor Control Research Laboratory, Department of Kinesiology and Community Health, University of Illinois at Urbana Champaign, Urbana, IL, United States; ^2^Illinois Multiple Sclerosis Research Collaborative, University of Illinois at Urbana Champaign, Urbana, IL, United States; ^3^Disability Participation & Quality of Life Research Laboratory, Department of Kinesiology and Community Health, University of Illinois at Urbana Champaign, Urbana, IL, United States

**Keywords:** seated postural control, smartphone technology, wheelchair users, clinical tests, measurement tool

## Abstract

Seated postural control is essential for wheelchair users to maintain proper position while performing activities of daily living. Clinical tests are commonly used to measure seated postural control, yet they are subjective and lack sensitivity. Lab-based measures are highly sensitive but are limited in scope and restricted to research settings. Establishing a valid, reliable, and accessible measurement tool of seated postural control is necessary for remote, objective assessments. Therefore, the purpose of this study was to examine the validity, reliability, and sensitivity of smartphone-based postural control assessments in wheelchair users. Eleven participants (age: 35.4 ± 17.9) completed two experimental visits 1-week apart consisting of three clinical tests: Trunk Control Test (TCT), Function in Sitting Test (FIST), and Tee-shirt Test, as well as, standardized instrumented balance tasks that manipulated vision (eyes open and closed), and trunk movement (functional reach and stability boundary). During these tasks, participants held a smartphone instrumented with a research-grade accelerometer to their chest. Maximum and root mean square (RMS) acceleration in the medial-lateral (ML) and anterior-posterior (AP) axes were derived. Participants were grouped into non-impaired and impaired postural groups based on FIST scores. Spearman rank-order correlations were conducted between the two devices' outcome measurements and between these measures and those of the clinical tests. Receiver operating characteristic (ROC) curves and the area under the curves (AUC) were determined to distinguish participants with and without impaired postural control. The reliability of outcome variables was assessed using inter-class correlations. Strong correlations between outputs derived from the smartphone and research-grade accelerometer were seen across balance tasks (ρ = −0.75–1.00; *p* ≤ 0.01). Numerous significant moderate correlations between clinical test outcomes and smartphone and research-grade RMS ML accelerometry were seen (ρ = −0.62 to 0.83 (*p* ≤ 0.044)]. On both devices, the AUC for ROC plots were significant for RMS ML sway during the eyes open task and functional stability boundary (*p* < 0.05). Reliability of smartphone accelerometry was comparable to the research-grade accelerometer and clinical tests. This pilot study illustrated that smartphone-based accelerometry may be able to provide a valid and reliable assessment of seated postural control and have the ability to distinguish between those with and without impaired postural control.

## Introduction

It is currently estimated that there are ~65 million wheelchair users worldwide (Physiopedia Contributors, 2018). Of these, ~3.3 million reside in the United States of America, where researchers are expecting annual growth of new users due to the exponential growth of older adults (Karmarkar et al., 2011). Wheelchair users face numerous challenges to maintaining an active and engaged life, which can be exacerbated by impaired seated postural control. Seated postural control is the ability to maintain one's center of mass within stability boundaries while in a seated position, and is comprised of a complex interplay of sensory processing and motor outputs (Ivanenko and Gurfinkel, 2018; Barbado et al., 2019). Alterations to sensory or motor processing can result in a decline in seated postural control (Shin and Sosnoff, 2013), and jeopardize an individual's ability to safely perform activities of daily living (Rice et al., 2015). As such, improving seated postural control is a common goal of rehabilitation interventions (Williams and Vette, 2019). Consequently, objectively measuring seated postural control in wheelchair users is necessary to guide prevention and rehabilitative strategies.

There are numerous ways to measure seated postural control. Researchers have developed several clinical measures including, but not limited to, the Function in Sitting Test (Gorman et al., 2010; Sung et al., 2016; Abou et al., 2019), Trunk Control Test (TCT) (Quinzaños et al., 2014), and the Tee-shirt Test (Boswell-Ruys et al., 2009) to assess seated postural control. These clinical measures have few technological requirements but require clinical expertise to perform. There are also concerns that these measures are subjective and lack sensitivity (Nguyen et al., 2012). Researchers have also utilized three-dimensional motion capture techniques (Murans et al., 2011; Curtis et al., 2015), video-based measurements (Sánchez et al., 2017), post-urography (Murans et al., 2011; Shin and Sosnoff, 2013), and research-grade accelerometers (Kim et al., 2018) to assess seated postural control. These research lab-based measures are objective and sensitive to impairment but require relatively expensive technology, expertise, and are limited to lab-based settings. Establishing an objective valid and reliable measurement tool to understand and monitor seated postural control is warranted (Rice et al., 2015).

A possible avenue for achieving objective accessible measures of seated postural control is through the utilization of mobile technology. Indeed, researchers have leveraged mobile health technology, specifically smartphone and tablet embedded sensors, to assess standing postural control (Roeing et al., 2017; Reyes et al., 2018). Recent work has shown that mobile technology is a valid (Cerrito et al., 2015; Hsieh et al., 2019) and reliable (Mellone et al., 2012; Cerrito et al., 2015) tool to provide objective assessments of standing balance, have a high level of usability (Hsieh et al., 2018) and are sensitive to impairment (Hsieh et al., 2019). Although promising, the validity, reliability, and sensitivity of smartphone-based seated postural control assessments in wheelchair users have not yet been investigated. Therefore, the purpose of the current pilot study is to determine the validity, reliability, and sensitivity of smartphone-based seated postural control assessments in adult wheelchair users, as an initial step in the remote monitoring of seated postural control. Based on previous standing balance research, we hypothesized that smartphone-based accelerometry can provide a valid and reliable measure of seated postural control and have the ability to distinguish between those with and without impaired postural control.

## Materials and Methods

### Participants

Eleven non-ambulatory adults (age: 35.4 ± 17.9 years; gender: 4 males, 7 females) were recruited from the local community to participate in the current study. To be eligible, individuals were required to be ≥18 years old, utilize a wheeled mobility device for their main form of mobility, manual dexterity sufficient to swipe on a smartphone, normal or corrected to normal hearing and vision, and able to read and speak English. Individuals were excluded from the study if they were unable to meet these criteria or if they were unable to sit upright for at least 1-h. The University of Illinois at Urbana-Champaign Institutional Review Board approved all procedures, and all participants provided written informed consent before engaging in research activities.

### Research Protocol and Data Analyses

**Figure 1:**
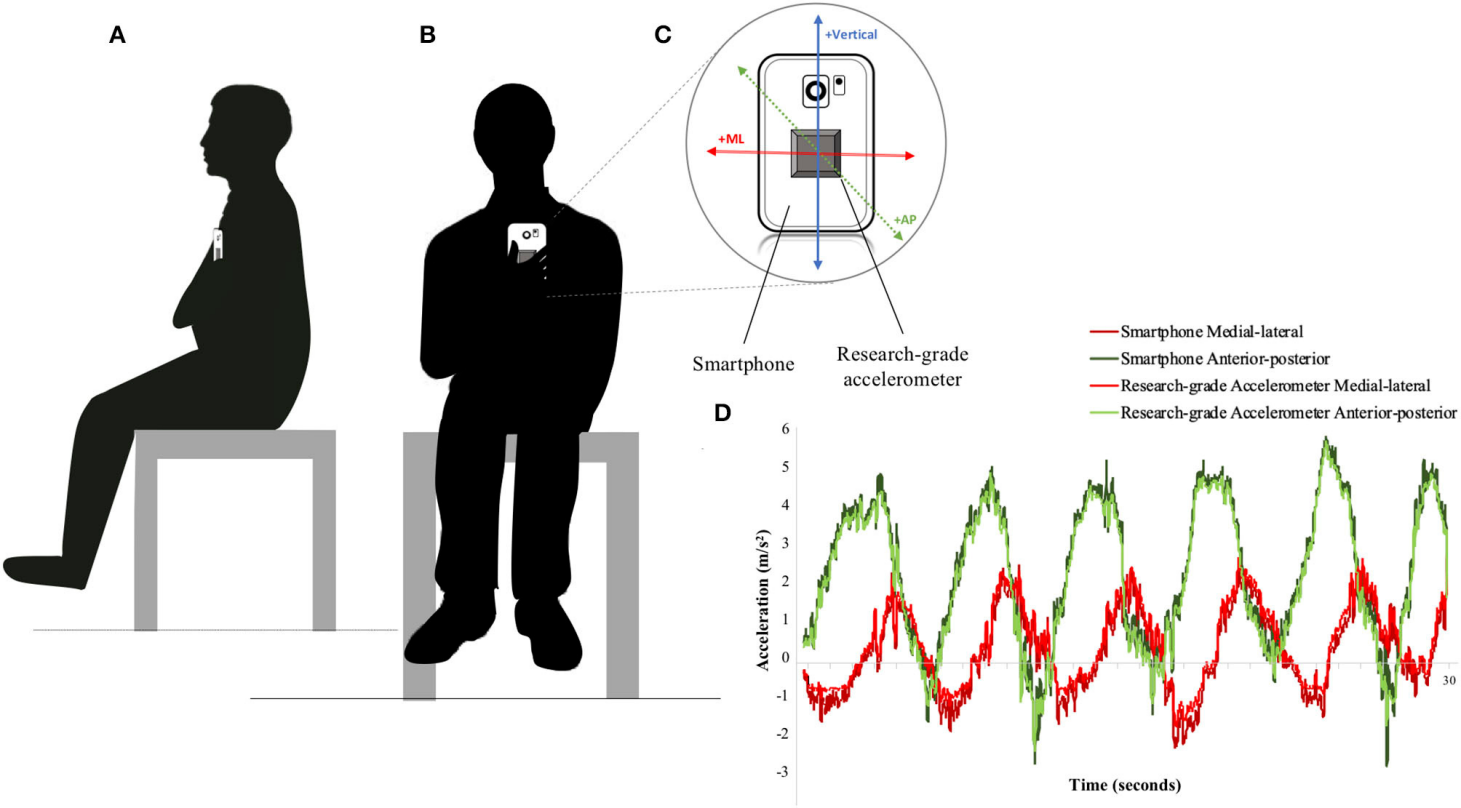
Illustration of two participants completing the seated postural control assessment. **(A)** represents a participant facing the side, **(B)** represents a participant facing forward, **(C)** depicts the research-grade accelerometer affixed to the back of the smartphone and the aligned axes orientation (ML, medial-lateral; AP, anterior-posterior), and **(D)** provides an example plot of accelerometry data from the smartphone and research-grade accelerometer, recorded during the Functional Stability Boundary Test.

Participants completed two identical experimental sessions, one week apart. The first experimental session began by obtaining written informed consent and participant demographic information. At each session, participants completed three clinical tests that have been shown to provide a valid measure of seated posture control: the Function in Sitting Test (FIST) (Abou et al., 2019), Trunk Control Test (TCT) (Quinzaños et al., 2014), and Tee-shirt Test (Boswell-Ruys et al., 2009). An individual's maximum forward and lateral reach distances (cm) were recorded during the FIST and also used as a clinical outcome, as they are an indication of seated balance (Lynch et al., 1998; Boswell-Ruys et al., 2009; Kalron et al., 2016). Following these tests, participants completed a series of unsupported seated balance tasks on a flat surface while holding a smartphone (Samsung Galaxy S6, Samsung, Seoul, South Korea) with a research-grade accelerometer affixed to the back with double-sided tape in a standardized position and orientation (The Opal, APDM Wearable Technologies, Portland, OR) (see Figure 1). In this arrangement, there were no gaps between the accelerometer and smartphone. Pilot testing informed the standardized alignment, positioning, and ensured a rigid adhesion of the research-grade accelerometer to the smartphone. This methodological approach is based in part on previous research examining the use of smartphones to access standing postural control (Hsieh et al., 2019).

Four seated balance tasks were completed in a standardized order that increased in difficulty: static sitting with eyes open (EO), static sitting with eyes closed (EC), functional reach (FR), and functional stability boundary (FSB). These tests were chosen because of their ability to provide insight into those with and without impaired postural control (Shin and Sosnoff, 2013).

All tests except for the functional reach task were completed for 30 s. The functional reach task was not constrained by time. Two trials of each task were completed. During EO, EC, and FSB balance tasks, participants held the smartphone with their dominant hand against their sternum and in standardized orientation (see Figure 1). This standardized orientation was also used during the functional reach task except for the phone being held in the participant's non-dominated hand. Proper holding and placement of the smartphone were modeled for the participants by the research staff. Consistency of alignment (anterior-posterior and medial-lateral axes) between the smartphone and participant were visually monitored by trained research personnel within and between testing sessions. If participants did not hold the phone correctly, trials were removed from analyses. With findings from the current study informing the development of a self-administered mobile health application, the methods of holding the smartphone in place of fixing it to the individual are imperative when accounting for “real-life” use and error. The smartphone was sampled at an average rate of 200 Hz and the research-grade accelerometer was collected at 128 Hz.

A custom MATLAB script (MathWorks Inc., Natick, MA) was used to downsample and temporally and spatially align (with respect to axes and gravity) all accelerometry data. Data were downsampled to 100 Hz and filtered using a 4th-order lowpass Butterworth filter set to 10 Hz. The time-series used for analysis was 30 s. Maximum (MAX) and root mean squared (RMS) acceleration time-series from each device along the anterior-posterior (AP) and medial-lateral (ML) axes, as well as the 95% confidence ellipse area (CEA), were calculated. These measures are seen to be a valid assessment of postural stability (Ozinga et al., 2015) and sensitive enough to identify impairment in other populations (Galán-Mercant and Cuesta-Vargas, 2014; Ozinga et al., 2015; Hsieh et al., 2019).

### Statistical Analysis

IBM Statistical Package for the Social Sciences (SPSS) for Windows, version 26 (IBM Corp, Armonk, NY) was used to complete all statistical analyses with statistical significance set at α = 0.05. All values, with exception of area under the curve, are reported as mean ± standard deviation. Extreme outliers, as defined as, any data values which lie more than 3.0 times the interquartile range were removed from the data set. Within the first and second sessions, extreme outliers made up 0.97 and 1.29% of the data, respectively. Once extreme outliers were removed, the two trials of each balance task (EO, EC, FR, and FSB) from a given session were averaged together. To assess the validity of the smartphone measurements, Spearman rank-order correlations between the smartphone and research-grade accelerometer were determined for all conditions. To understand the validity of these measurements in assessing seated postural control, Spearman rank-order correlations were completed between the smartphone and research-grade accelerometer to clinical measure outcomes. Correlation coefficients of 0.1 were considered small, 0.3 were considered moderate, and 0.5 were considered large (Cohen et al., 2003). The reliability of the smartphone, research-grade accelerometer, and clinical tests were measured by conducting interclass correlations (ICC) of their respective outcome variables from session 1 and session 2. Values <0.5 were considered poor, between 0.5 and 0.75 were considered moderate, between 0.75 and 0.9 were considered good, and >0.90 were indicative of excellent reliability (Koo and Li, 2016).

A median split of FIST scores was used to separate participants into two groups: those with and without impaired seated postural control. Once separated, independent sample *T*-tests were performed to identify potential differences in age and clinical test outcome measures during all balance conditions. To further understand the difference between the two groups, the effect sizes (Cohen's *d*) were calculated. Effect sizes were classified as small (*d* = 0.20), medium (*d* = 0.50), and large (*d* = 0.80) (Jacob, 1988). To determine the sensitivity of the smartphone and research-grade accelerometer, receiving operating characteristic (ROC) curves were constructed and the area under the curve (AUC) was calculated for RMS ML, RMS AP, and CEA to determine the classification accuracy of those with and without impaired seated postural control.

## Results

Results from the research grade accelerometer indicated that MAX ML acceleration ranged from −6.92 to 5.47 m/s^2^ and had mean value of −0.68 ± 2.16 m/s^2^, and MAX AP acceleration ranged from 0.68 to 8.66 m/s^2^ and had mean value of 5.37 ± 2.09 m/s^2^. RMS ML acceleration ranged from 0.22 to 3.98 m/s^2^ and had mean value of 1.16 ± 0.86 m/s^2^, and RMS AP acceleration ranged from 0.24 to 7.47 m/s^2^ and had mean value of 4.13 ± 1.87 m/s^2^.

As for the smartphone, MAX ML acceleration ranged from −0.99 to 6.71 m/s^2^ and had mean value of 1.79 ± 1.64 m/s^2^, and MAX AP acceleration ranged from −8.28 to 7.46 m/s^2^ and had mean value of 2.11 ± 3.13 m/s^2^. RMS ML acceleration ranged from 0.16 to 4.16 m/s^2^ and had mean value of 1.18 ± 0.88 m/s^2^, and RMS AP acceleration ranged from 0.39 to 7.73 m/s^2^ and had mean value of 4.31 ± 1.90 m/s^2^.

### Validity

Spearman rank-order correlations between the smartphone and research-grade accelerometer outcome variables revealed numerous significant relations. Maximum acceleration along the ML (EO and EC) (*p* ≤ 0.01) and AP (EO, EC, and FR) (*p* ≤ 0.01) axes were significantly correlated between devices (see Table 1). Measures of RMS acceleration and CEA yielded strong, significant correlations between the two devices (*p* ≤ 0.011), except for ML acceleration during the eyes closed balance task (*p* = 0.124) (see Table 1).

**Table 1:** Presents the correlations (Rho) of maximum (MAX) and root mean square (RMS) acceleration as derived through smartphone and research-grade accelerometry.

**Balance task**	**Accelerometry variable**	**Rho (ρ)**	***p*-value**
Eyes open	MAX ML	0.755	<0.01**
	MAX AP	0.982	<0.01**
	RMS ML	0.918	<0.01**
	RMS AP	1.000	<0.01**
	CEA	0.727	0.011*
Eyes closed	MAX ML	0.866	0.01**
	MAX AP	0.864	0.01**
	RMS ML	0.492	0.124
	RMS AP	0.936	<0.01**
	CEA	0.909	<0.01**
Functional reach	MAX ML	0.515	0.128
	MAX AP	0.818	<0.01**
	RMS ML	0.964	<0.01**
	RMS AP	0.945	<0.01**
	CEA	0.982	<0.01**
Functional stability boundary	MAX ML	0.218	0.519
	MAX AP	0.527	0.096
	RMS ML	0.991	<0.01**
	RMS AP	1.000	<0.01**
	CEA	0.891	<0.01**

** *A significant correlation where p ≤ 0.01 level (2-tailed)*.

* *p < 0.05. Red coloring indicates a small correlation coefficient, yellow indicates moderate, and green indicates large*.

Spearman rank-order correlations were also completed between clinical test outcomes and smartphone and research-grade accelerometer outcome variables during the seated balance tasks (i.e., EO, EC, FR, and FSB). Overall, numerous significant correlations were seen between the clinical tests and RMS accelerometry on both devices in the ML axis (see Supplementary Material). Smartphone and research-grade RMS ML accelerometry yielded strong, positive, significant correlations with the clinical tests. Rho values from smartphone accelerometry ranged from 0.642 (*p* = 0.033) to 0.807 (*p* ≤ 0.01) (see Table 2), while research-grade accelerometry ranged from 0.615 (*p* = 0.044) to 0.826 (*p* ≤ 0.01). The TCT was the only clinical test to not display a significant association with RMS ML acceleration (see Table 2).

**Table 2:** Presents the correlations (Rho) between clinical test outcomes and root mean square (RMS) acceleration as derived through smartphone and research-grade accelerometry.

**Device**	**Clinical test**	**Balance task**	**Accelerometry variable**	**Rho (*p*)**	***p*-value**
Smartphone	Function in sitting test	Functional stability boundary	RMS ML	0.717	0.013*
	Tee-shirt test	Eyes closed	RMS ML	0.642	0.033*
		Functional reach	RMS ML	0.718	0.013*
	Forward reach	Eyes open	RMS ML	0.788	0.004*
		Eyes closed	RMS ML	0.665	0.026*
		Functional stability boundary	RMS ML	0.651	0.030*
	Lateral reach	Functional stability boundary	RMS ML	0.807	<0.01**
Research-grade accelerometer	Function in sitting test	Eyes open	RMS ML	0.621	0.041*
		Eyes closed	RMS ML	0.621	0.041*
		Functional stability boundary	RMS ML	0.685	0.020*
	Tee-shirt test	Functional reach	RMS ML	0.618	0.043*
	Forward reach	Eyes open	RMS ML	0.615	0.044*
		Eyes closed	RMS ML	0.615	0.044*
		Functional stability boundary	RMS ML	0.633	0.036*
	Lateral reach	Eyes open	RMS ML	0.706	0.015*
		Eyes closed	RMS ML	0.706	0.015*
		Functional stability boundary	RMS ML	0.826	<0.01**

** *A significant correlation where p ≤ 0.01 level (2-tailed)*.

* *p < 0.05. Green coloring indicates a large correlation coefficient*.

### Reliability

Reliability was seen across all clinical measurements (*p* ≤ 0.005), except the TCT (*p* = 0.077) (see Table 3). As for accelerometry, 55% of the smartphone and 70% of research-grade accelerometer outcome variables were found to be reliable (*p* < 0.05) (see Table 4). The smartphone was the most reliable across outcome variables during the EC balance test, while the research-grade accelerometer was equally reliable during the EC and FSB tests (see Table 4).

**Table 3:** Interclass correlations (ICC) between clinical test outcomes during session 1 and session 2.

**Clinical test**	**95% CI**	**ICC**	***p*-value**
FIST	[0.296, 0.924]	0.745	<0.01**
TCT	[−0.185, 0.810]	0.438	0.077
Tee-shirt test	[0.346, 0.932]	0.769	<0.01**
Forward reach	[0.234, 0.914]	0.714	<0.01**
Lateral reach	[0.404, 0.940]	0.795	<0.01**

*Function in sitting test: FIST, Trunk Control Test: TCT*.

** *A significant correlation where p < 0.01. Red coloring indicates poor reliability, blue indicates moderate reliability, yellow indicates good reliability, and green indicates excellent reliability*.

**Table 4:** Interclass correlations (ICC) between maximum (MAX) acceleration, root mean squared (RMS) acceleration, and confidence ellipse area (CEA) as recorded through smartphone and research-grade accelerometry during session 1 and session 2.

**Device**	**Balance tasks**	**Accelerometry variable**	**95% CI**	**ICC**	***p*-value**
Smartphone	Eyes open	MAX ML	[−0.378, 0.723]	0.253	0.214
		MAX AP	[−0.168, 0.815]	0.451	0.070
		RMS ML	[−0.208, 0.801]	0.418	0.088
		RMS AP	[0.885, 0.991]	0.968	<0.01**
		CEA	[−0.593, 0.558]	−0.026	0.532
	Eyes closed	MAX ML	[−0.005, 0.864]	0.572	0.026*
		MAX AP	[−0.029, 0.877]	0.583	0.030*
		RMS ML	[0.170, 0.902]	0.679	<0.01**
		RMS AP	[0.947, 0.996]	0.986	<0.01**
		CEA	[−0.638, 0.507]	−0.098	0.619
	Functional reach	MAX ML	[0.311, 0.927]	0.752	<0.01**
		MAX AP	[−0.419, 7.38]	0.244	0.234
		RMS ML	[0.734, 0.978]	0.921	<0.01**
		RMS AP	[0.659, 0.971]	0.895	<0.01**
		CEA	[−0.576, 0.576]	0.000	0.500
	Functional stability boundary	MAX ML	[0.123, 0.893]	0.653	0.011*
		MAX AP	[−0.288, 0.769]	0.346	0.136
		RMS ML	[0.747, 0.979]	0.925	<0.01**
		RMS AP	[0.765, 0.981]	0.931	<0.01**
		CEA	[−0.249, 0.785]	0.382	0.110
Research-grade accelerometer	Eyes Open	MAX ML	[−0.572, 0.580]	0.006	0.493
		MAX AP	[0.860, 0.989]	0.960	<0.01**
		RMS ML	[0.074, 0.899]	0.647	0.016*
		RMS AP	[0.891, 0.992]	0.969	<0.01**
		CEA	[−0.609, 0.541]	−0.050	0.562
	Eyes Closed	MAX ML	[0.127, 0.894]	0.655	<0.01**
		MAX AP	[0.762, 0.981]	0.930	<0.01**
		RMS ML	[0.074, 0.899]	0.647	0.016*
		RMS AP	[0.891, 0.992]	0.969	<0.01**
		CEA	[−0.576, 0.576]	0.000	0.500
	Functional reach	MAX ML	[−0.608, 0.596]	−0.009	0.511
		MAX AP	[0.520, 0.955]	0.844	<0.01**
		RMS ML	[0.658, 0.971]	0.895	<0.01**
		RMS AP	[0.616, 0.966]	0.880	<0.01**
		CEA	[−0.576, 0.576]	0.000	0.500
	Functional stability boundary	MAX ML	[0.352, 0.933]	0.772	<0.01**
		MAX AP	[0.288, 0.923]	0.741	<0.01**
		RMS ML	[0.744, 0.979]	0.924	<0.01**
		RMS AP	[0.780, 0.982]	0.936	<0.01**
		CEA	[−0.502, 0.641]	0.104	0.374

**A significant correlation where p < 0.01 while *p < 0.05. Red coloring indicates poor reliability, blue indicates moderate reliability, yellow indicates good reliability, and green indicates excellent reliability.

### Sensitivity

To determine sensitivity, the 11 participants were separated into two groups, those with (*n* = 5) and without (*n* = 6) impaired seated postural control (see Table 5). Per design, group differences were observed in the FIST (*p* = 0.009) as well as the TCT performance (*p* = 0.023). The effect sizes ranged from small to large (*d*: −0.40 to −2.59).

**Table 5:** Participant demographic information.

	**Participants with impaired postural stability**	**Participants without impaired postural stability**	**Levene's test for equality of variances**	**Independent samples test (2-tailed)**	**95% CI**	**Cohen's *d***
Sample size	*n* = 5	*n* = 6	–	–	–	–
Age (years)	27.8 ± 10.9	41.7 ± 21.0	0.141	0.217	[−9.78, 37.5]	−0.947
Gender	Males: 2, Females:3	Males: 2, Females: 4	–	–	–	–
Reason for wheeled- mobility	SCI: 3, Sacral Agenesis: 1, diastematomyelia: 1	SCI: 2, MS: 3, CP: 1	–	–	–	–
FIST	41.4 ± 6.0	53.2 ± 2.4	0.031*	<0.01**	[4.42, 19.1]	−2.585
TCT	17.2 ± 3.0	21.2 ± 1.7	0.064	0.023*	[0.686, 7.25]	−1.575
Tee-shirt test (sec)	20.8 ± 7.6	25.3 ± 20.9	0.073	0.659	[−17.9, 27.0]	−0.404
Forward reach (cm)	10.6 ± 4.9	18.6 ± 11.1	0.042*	0.157	[−3.92, 19.9]	−1.114
Lateral reach (cm)	4.8 ± 3.1	10.7 ± 7.0	0.357	0.116	[−1.78, 13.5]	−1.274

Values are reported as mean ± standard deviation. **p < 0.01, while *p < 0.05.

**Figure 2:**
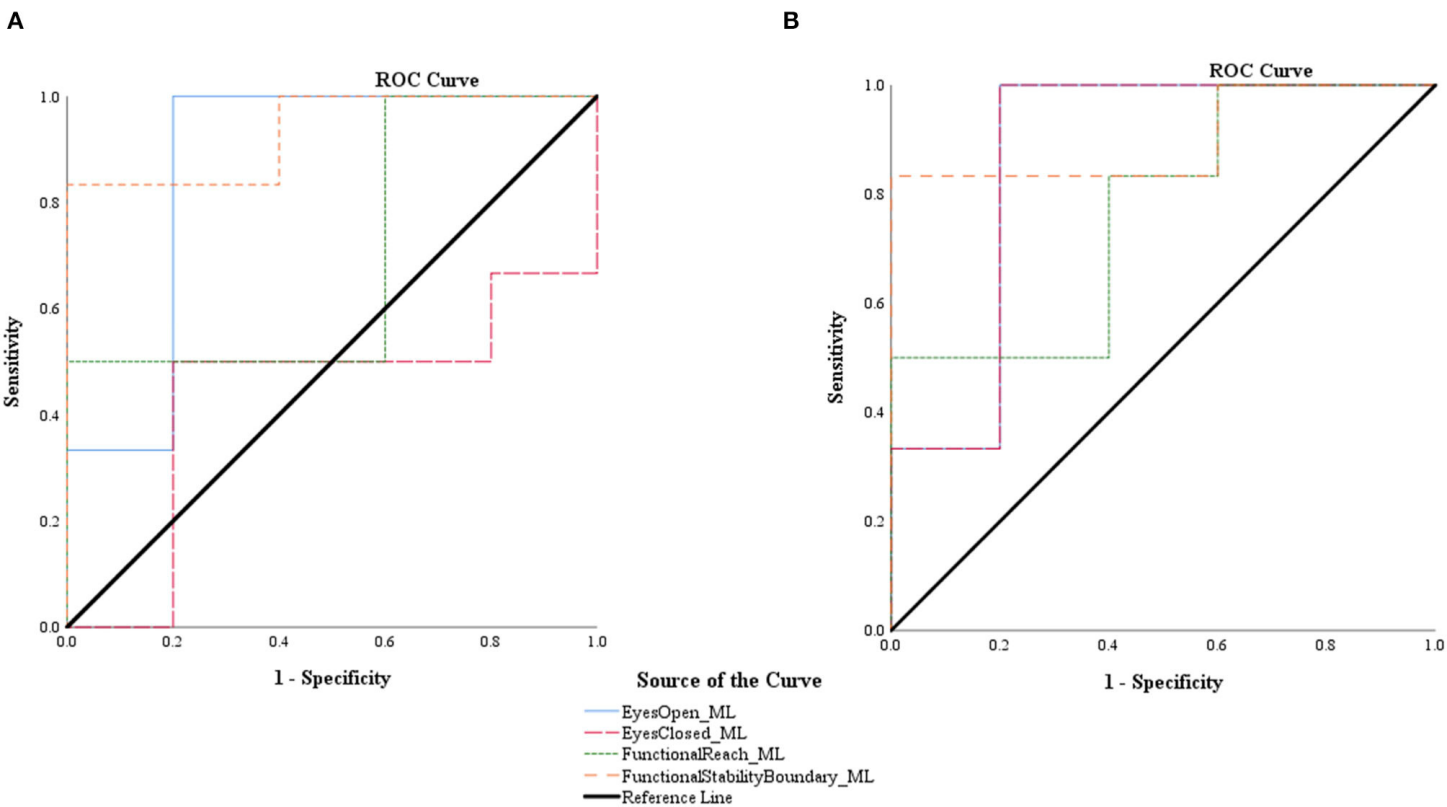
Receiver operating characteristic (ROC) curves for root mean squared smartphone **(A)** and research-grade accelerometer **(B)** acceleration in the medial-lateral (ML) direction during all balance tasks.

To distinguish individuals with and without impaired seated postural control, ROC curves were constructed, and AUC was calculated for smartphone and research-grade accelerometry RMS ML, RMS AP, and CEA (see Table 6). The AUC for smartphone RMS ML ranged from 0.433 ± 0.188 to 0.933 ± 0.078, RMS AP ranged from 0.500 ± 0.186 to 0.667 ± 0.174, and CEA ranged from 0.467 ± 0.209 to 0.800 ± 0.144 (values are mean ± SE). Research-grade accelerometry yielded similar findings with the AUC for RMS ML ranging from 0.767 ± 0.149 to 0.900 ± 0.104, RMS AP ranging from 0.500 ± 0.186 to 0.600 ± 0.181, and CEA ranging from 0.367 ± 0.182 to 0.833 ± 0.128 (values are mean ± SE). The AUC was statistically significant for smartphone and research-grade accelerometry RMS ML sway during the EO (*p* = 0.045, both) and FSB (*p* = 0.018 and 0.028, respectively) balance tasks (see Table 6 and Figure 2). The AUC was also statistically significant for research-grade accelerometry RMS ML sway during the EC balance task (*p* = 0.045).

**Table 6:** Receiver operating characteristic curve statistical outcomes.

**Device**	**Balance task**	**Accelerometry variable**	**AUC (SE)**	***p*-value**	**95% Confidence Interval**
Smartphone	Eyes open	RMS ML	0.867 (0.130)	0.045*	[0.612, 1.000]
		RMS AP	0.600 (0.181)	0.584	[0.245, 0.955]
		CEA	0.500 (0.207)	1.000	[0.094, 0.906]
	Eyes closed	RMS ML	0.433 (0.188)	0.715	[0.065, 0.801]
		RMS AP	0.667 (0.174)	0.361	[0.325, 1.000]
		CEA	0.467 (0.209)	0.855	[0.094, 0.906]
	Functional reach	RMS ML	0.700 (1.70)	0.273	[0.366, 1.000]
		RMS AP	0.533 (0.189)	0.855	[0.164, 0.903]
		CEA	0.500 (0.187)	1.000	[0.133, 0.867]
	Functional stability boundary	RMS ML	0.933 (0.078)	0.018*	[0.780, 1.000]
		RMS AP	0.500 (0.186)	1.000	[0.134, 0.866]
		CEA	0.800 (0.144)	0.100	[0.518, 1.000]
Research-grade accelerometer	Eyes open	RMS ML	0.867 (0.130)	0.045*	[0.612, 1.000]
		RMS AP	0.600 (0.181)	0.584	[0.245, 0.955]
		CEA	0.400 (0.201)	0.584	[0.005, 0.795]
	Eyes closed	RMS ML	0.867 (0.130)	0.045*	[0.612, 1.000]
		RMS AP	0.600 (0.181)	0.584	[0.245, 0.955]
		CEA	0.367 (0.182)	0.465	[0.011, 0.723]
	Functional reach	RMS ML	0.767 (0.149)	0.144	[0.474, 1.000]
		RMS AP	0.500 (0.192)	1.000	[0.123, 0.877]
		CEA	0.533 (0.189)	0.855	[0.164, 0.903]
	Functional stability boundary	RMS ML	0.900 (0.104)	0.028*	[0.697, 1.000]
		RMS AP	0.500 (0.186)	1.000	[0.134, 0.866]
		CEA	0.833 (0.128)	0.068	[0.583, 1.000]

*AUC, area under the curve; SE, standard error*.

* *p < 0.05*.

## Discussion

Understanding the validity, reliability, and sensitivity of smartphone-based seated postural control during various balance tasks is critical in the efforts of providing wheelchair users with an objective and accessible tool to measure seated postural control. Within the current study, smartphone-based measures of seated postural control were found to be valid, have reliability that was on par or greater than the clinical tests, and capable of discriminating between individuals with and without impaired seated postural control. Collectively, the observations provide preliminary evidence that smartphone-based accelerometry is suitable for objectively measuring seated postural control in adult wheelchair users.

Due to the strong significant correlations between outputs derived from the smartphone and research-grade accelerometer, the current investigation indicates that the smartphone-based accelerometry is potentially as valid as a research-grade accelerometer. This is in line with recent studies which illustrated that smartphone accelerometry provided a valid measure of standing postural stability when compared against research-grade equipment (Cerrito et al., 2015; Hsieh et al., 2019).

Quantifying seated postural control has been a topic of scientific interest, utilizing a wide array of technology (e.g., three-dimensional motion capture, video-based measures, and force plate measures; Murans et al., 2011; Shin and Sosnoff, 2013; Curtis et al., 2015; Sánchez et al., 2017). Accelerometry has been used to evaluate the movement of transfers in adult wheelchair users (Barbareschi et al., 2018), yet limited work has utilized this technology to quantify seated postural control, resulting in limited recommendations concerning how to best quantify the acceleration signal. Research focusing on standing balance has recommended the use of RMS as the “best” measure (Kosse et al., 2015; Ozinga et al., 2015; Hsieh et al., 2019). Consistent with these recommendations, the current investigation observed numerous strong correlations between smartphone and research-grade accelerometry when RMS quantified the signal. Few significant relationships were observed between MAX measures of acceleration. This is in agreement with the discrepancy in MAX accelerometry ranges presented from each device. Similar findings were also seen when assessing the relationship between smartphone accelerometry and clinical measure outcomes. Collectively this supports the notion that RMS of acceleration is a valid measure of seated postural control and should be incorporated into future study designs investigating accelerometry-based seated postural control.

Along with identifying the validity of accelerometry-based movement and balance tasks, past investigations have shown this form of technology to be reliable (Mellone et al., 2012; Cerrito et al., 2015; Kosse et al., 2015; Silsupadol et al., 2017; Douma et al., 2018). Consistent with previous research, findings from the current study indicate that the RMS of smartphone acceleration is as reliable as that of a research-grade accelerometer.

Clinical tests, particularly the FIST and TCT, have been reported as reliable measures of seated postural control in clinical populations (Quinzaños et al., 2014; Abou et al., 2019). The current results confirm the reliability of the FIST, Tee-shirt Test, Forward Reach, and Lateral Reach. The level of reliability of smartphone-based accelerometry was on par with or greater than those of the clinical tests. Such observations further support the notion that smartphone technology is a reliable and objective measurement of seated postural control for wheelchair users.

In order to provide meaningful results, smartphone technology must have the sensitivity to differentiate between those with varying degrees of postural control. In the past, smartphone accelerometry has been able to do discriminate between standing postural control in frailty (frail/non-frail) (Galán-Mercant and Cuesta-Vargas, 2014) and fall risk (Hsieh et al., 2019) within older adults. Within the current study, measurements from both devices during the easiest (EO) and most challenging (FSB) balance tasks were able to identify participants with and without impaired postural control, specifically in the ML direction. This is further supported by the numerous strong, positive, significant correlations between RMS ML smartphone and research-grade accelerometry and clinical measure outcomes. Although ML postural control has been implicated in impairment in standing balance (Sosnoff et al., 2011), we believe this is one of the first investigations to highlight this in seated postural control. Recent work also supports this observation by providing evidence that those with impaired seated postural control exhibit greater decrements in their lateral (ML) reach than forward (AP) reach (Abou et al., 2019). These collective findings indicate that smartphone technology may have the sensitivity to identify those with and without impaired seated postural control and that postural instability within wheelchair users may be rooted in mediolateral instability.

## Conclusion

To better understand seated postural control, and monitor changes over time, we must establish an objective and sensitive measurement tool. To our knowledge, this is the first investigation examining the validity, reliability, and sensitivity of smartphone-based accelerometry as a tool to quantify seated postural control in adult wheelchair users. Results from this study illustrated that smartphone technology may be able to provide a valid and reliable assessment of seated postural control and have the ability to distinguish between those with and without impaired postural control—especially in the ML plane. Given the ubiquitous nature of smartphones in society, there is great potential for mobile technology to provide quick, easily accessible, and objective remote monitoring of seated postural control in adult wheelchair users.

## Limitations and Future Directions

Limitations of the current study include limited sample size, albeit with a diverse range of seated postural control, the use of a single smartphone and research-grade accelerometer, and the potential introduction of motion artifact with the use of a handheld accelerometer. Future research should incorporate a larger sample to assess the relationship between clinical scores and accelerometry, further investigate the reliability of accelerometry based seated postural control assessments and the feasibility of leveraging this form of technology for remote assessment, and examine the difference between a sensor worn on the body and a handheld sensor. Along with this, researchers need to develop a health application interface to provide this type of assessment and determine its usability, validity, and reliability of results, responsiveness to interventions (i.e., sensitivity to changes in seated balance), and home use acceptance.

## Data Availability Statement

The datasets generated for this study are available on request to the corresponding author.

## Ethics Statement

The studies involving human participants were reviewed and approved by the University of Illinois at Urbana-Champaign Office for the Protection of Research Subjects. The patients/participants provided their written informed consent to participate in this study.

## Author Contributions

LR and JS: conceptualization and funding acquisition. MF and LA: data collection. MF: organized database, software coding, and statistical analysis. JS: provided resources and project administration. MF and JS: writing—original draft. All authors contributed to the methodology, manuscript revision, and read and approved the submitted version.

## Conflict of Interest

JS has partial ownership in Sosnoff Technologies, LLC a company that may be affected by the research reported in the enclosed paper. This conflict of interest is managed by a plan approved by the University of Illinois at Urbana-Champaign. The remaining authors declare that the research was conducted in the absence of any commercial or financial relationships that could be construed as a potential conflict of interest.
