# Supplementary material for: The Validity, Reliability, and Sensitivity of a Smartphone-Based Seated Postural Control Assessment in Wheelchair Users: A Pilot Study
Source: Front Sports Act Living. 2020 Dec 17;2:540930. doi: 10.3389/fspor.2020.540930 (PMC7750873; doi:10.3389/fspor.2020.540930)
Supplement: Supplementary file 1 [file Data_Sheet_1.PDF]

Presents the correlations (Rho) between clinical test outcomes and maximum (MAX) and root mean square (RMS) acceleration as derived through smartphone accelerometry. ** indicates a significant correlation where $p \leq 0.01$ level (2-tailed). * represents that $p<0.05$. Red coloring indicates a small correlation coefficient, yellow indicates moderate, and green indicates large.

| Clinical Test | Balance Task | Accelerometry Variable | Rho (ρ) | *p*-value |
|---|---|---|---|---|
| Function In Sitting Test | Eyes Open | MAX ML | 0.224 | 0.508 |
| | | MAX Vertical | -0.237 | 0.482 |
| | | MAX AP | 0.210 | 0.535 |
| | | RMS ML | 0.548 | 0.081 |
| | | RMS Vertical | -0.269 | 0.423 |
| | | RMS AP | 0.205 | 0.544 |
| | | CEA | -0.192 | 0.572 |
| | Eyes Closed | MAX ML | 0.034 | 0.920 |
| | | MAX Vertical | -0.429 | 0.188 |
| | | MAX AP | 0.447 | 0.168 |
| | | RMS ML | -0.071 | 0.836 |
| | | RMS Vertical | -0.470 | 0.144 |
| | | RMS AP | 0.402 | 0.221 |
| | | CEA | -0.224 | 0.508 |
| | Functional Reach | MAX ML | -0.539 | 0.087 |
| | | MAX Vertical | -0.288 | 0.391 |
| | | MAX AP | -0.152 | 0.674 |
| | | RMS ML | 0.151 | 0.658 |
| | | RMS Vertical | 0.347 | 0.296 |
| | | RMS AP | -0.041 | 0.905 |
| | | CEA | -0.356 | 0.282 |
| | Funcational Stability Boundary | MAX ML | 0.114 | 0.738 |
| | | MAX Vertical | -0.087 | 0.800 |
| | | MAX AP | -0.105 | 0.759 |
| | | RMS ML | 0.717 | 0.013* |
| | | RMS Vertical | -0.260 | 0.440 |
| | | RMS AP | 0.123 | 0.718 |
| | | CEA | 0.374 | 0.257 |
| Trunk Control Test | Eyes Open | MAX ML | -0.194 | 0.568 |
| | | MAX Vertical | -0.078 | 0.819 |
| | | MAX AP | 0.041 | 0.904 |
| | | RMS ML | 0.290 | 0.386 |
| | | RMS Vertical | -0.092 | 0.788 |
| | | RMS AP | 0.014 | 0.968 |
| | | CEA | -0.475 | 0.140 |
| | Eyes Closed | MAX ML | -0.323 | 0.332 |
| | | MAX Vertical | -0.332 | 0.319 |
| | | MAX AP | 0.313 | 0.348 |
| | | RMS ML | -0.296 | 0.377 |
| | | RMS Vertical | -0.341 | 0.305 |
| | | RMS AP | 0.235 | 0.487 |
| | | CEA | -0.263 | 0.435 |
| | Functional Reach | MAX ML | -0.719 | 0.013* |
| | | MAX Vertical | 0.028 | 0.936 |
| | | MAX AP | -0.142 | 0.696 |
| | | RMS ML | -0.157 | 0.645 |
| | | RMS Vertical | 0.604 | 0.049* |
| | | RMS AP | -0.074 | 0.829 |
| | | CEA | -0.327 | 0.326 |
| | Functional Stability Boundary | MAX ML | -0.055 | 0.872 |
| | | MAX Vertical | 0.060 | 0.861 |
| | | MAX AP | -0.226 | 0.504 |
| | | RMS ML | 0.350 | 0.291 |
| | | RMS Vertical | 0.069 | 0.840 |
| | | RMS AP | 0.018 | 0.957 |
| | | CEA | 0.309 | 0.356 |
| Tee-shirt Test | Eyes Open | MAX ML | 0.145 | 0.670 |
| | | MAX Vertical | 0.191 | 0.574 |
| | | MAX AP | -0.264 | 0.433 |
| | | RMS ML | 0.227 | 0.502 |
| | | RMS Vertical | 0.300 | 0.370 |
| | | RMS AP | -0.336 | 0.312 |
| | | CEA | 0.245 | 0.467 |
| | Eyes Closed | MAX ML | 0.460 | 0.154 |
| | | MAX Vertical | 0.245 | 0.467 |
| | | MAX AP | -0.364 | 0.272 |
| | | RMS ML | 0.642 | 0.033* |
| | | RMS Vertical | 0.227 | 0.502 |
| | | RMS AP | -0.382 | 0.247 |
| | | CEA | 0.073 | 0.832 |
| | Functional Reach | MAX ML | 0.536 | 0.089 |
| | | MAX Vertical | 0.300 | 0.370 |
| | | MAX AP | -0.236 | 0.511 |
| | | RMS ML | 0.718 | 0.013* |
| | | RMS Vertical | 0.127 | 0.709 |
| | | RMS AP | 0.055 | 0.873 |
| | | CEA | 0.600 | 0.051 |
| | Funcational Stability Boundary | MAX ML | 0.409 | 0.212 |
| | | MAX Vertical | -0.073 | 0.832 |
| | | MAX AP | -0.373 | 0.259 |
| | | RMS ML | 0.218 | 0.519 |
| | | RMS Vertical | 0.027 | 0.937 |
| | | RMS AP | -0.318 | 0.34 |
| | | CEA | -0.255 | 0.45 |
| Forward Reach | Eyes Open | MAX ML | 0.506 | 0.113 |
| | | MAX Vertical | -0.592 | 0.055 |
| | | MAX AP | 0.560 | 0.073 |
| | | RMS ML | 0.788 | <0.01** |
| | | RMS Vertical | -0.533 | 0.091 |
| | | RMS AP | 0.460 | 0.154 |
| | | CEA | -0.255 | 0.449 |
| | Eyes Closed | MAX ML | 0.432 | 0.185 |
| | | MAX Vertical | -0.720 | 0.013* |
| | | MAX AP | 0.692 | 0.018* |
| | | RMS ML | 0.290 | 0.387 |
| | | RMS Vertical | -0.697 | 0.017* |
| | | RMS AP | 0.665 | 0.026* |
| | | CEA | -0.383 | 0.245 |
| | Functional Reach | MAX ML | -0.214 | 0.527 |
| | | MAX Vertical | -0.301 | 0.369 |
| | | MAX AP | 0.474 | 0.166 |
| | | RMS ML | 0.474 | 0.141 |
| | | RMS Vertical | -0.146 | 0.669 |
| | | RMS AP | 0.451 | 0.164 |
| | | CEA | 0.096 | 0.78 |
| | Functional Stability Boundary | MAX ML | 0.050 | 0.884 |
| | | MAX Vertical | -0.497 | 0.120 |
| | | MAX AP | 0.323 | 0.332 |
| | | RMS ML | 0.651 | 0.030* |
| | | RMS Vertical | -0.478 | 0.137 |
| | | RMS AP | 0.401 | 0.222 |
| | | CEA | 0.328 | 0.325 |
| Lateral Reach | Eyes Open | MAX ML | 0.743 | <0.01** |
| | | MAX Vertical | -0.413 | 0.207 |
| | | MAX AP | 0.440 | 0.175 |
| | | RMS ML | 0.752 | <0.01** |
| | | RMS Vertical | -0.431 | 0.185 |
| | | RMS AP | 0.376 | 0.254 |
| | | CEA | 0.055 | 0.872 |
| | Eyes Closed | MAX ML | 0.543 | 0.085 |
| | | MAX Vertical | -0.376 | 0.254 |
| | | MAX AP | 0.367 | 0.267 |
| | | RMS ML | 0.423 | 0.195 |
| | | RMS Vertical | -0.431 | 0.185 |
| | | RMS AP | 0.459 | 0.156 |
| | | CEA | 0.101 | 0.768 |
| | Functional Reach | MAX ML | -0.028 | 0.936 |
| | | MAX Vertical | -0.661 | 0.027* |
| | | MAX AP | 0.255 | 0.476 |
| | | RMS ML | 0.495 | 0.121 |
| | | RMS Vertical | -0.303 | 0.365 |
| | | RMS AP | 0.266 | 0.429 |
| | | CEA | 0.11 | 0.747 |
| | Functional Stability Boundary | MAX ML | 0.413 | 0.207 |
| | | MAX Vertical | -0.101 | 0.768 |
| | | MAX AP | 0.229 | 0.497 |
| | | RMS ML | 0.807 | <0.01** |
| | | RMS Vertical | -0.651 | 0.030* |
| | | RMS AP | 0.385 | 0.242 |
| | | CEA | 0.477 | 0.138 |
