# Supplementary material for: The Validity, Reliability, and Sensitivity of a Smartphone-Based Seated Postural Control Assessment in Wheelchair Users: A Pilot Study
Source: Front Sports Act Living. 2020 Dec 17;2:540930. doi: 10.3389/fspor.2020.540930 (PMC7750873; doi:10.3389/fspor.2020.540930)
Supplement: Supplementary file 2 [file Data_Sheet_2.PDF]

Presents the correlations (Rho) between clinical test outcomes and maximum (MAX) and root mean square (RMS) acceleration as derived through research-grade accelerometry. ** indicates a significant correlation where $p \leq 0.01$ level (2-tailed). * represents that $p<0.05$. Red coloring indicates a small correlation coefficient, yellow indicates moderate, and green indicates large.

Clinical Test	Balance Task	Accelerometry Variable	Rho (ρ)	*p*-value
**Function In Sitting Test**	Eyes Open	MAX ML	0.361	0.276
		MAX Vertical	-0.237	0.482
		MAX AP	0.205	0.544
		RMS ML	0.621	0.041*
		RMS Vertical	-0.205	0.544
		RMS AP	0.205	0.544
		CEA	-0.260	0.440
	Eyes Closed	MAX ML	0.205	0.544
		MAX Vertical	-0.429	0.188
		MAX AP	0.219	0.517
		RMS ML	0.621	0.041*
		RMS Vertical	-0.205	0.544
		RMS AP	0.205	0.544
		CEA	-0.457	0.158
	Functional Reach	MAX ML	-0.299	0.402
		MAX Vertical	-0.187	0.581
		MAX AP	-0.201	0.554
		RMS ML	0.247	0.465
		RMS Vertical	0.114	0.738
		RMS AP	-0.110	0.748
		CEA	-0.320	0.338
	Functional Stability Boundary	MAX ML	-0.397	0.226
		MAX Vertical	-0.100	0.769
		MAX AP	0.329	0.324
		RMS ML	0.685	0.020*
		RMS Vertical	-0.260	0.440
		RMS AP	0.123	0.718
		CEA	0.447	0.168
**Trunk Control Test**	Eyes Open	MAX ML	0.005	0.989
		MAX Vertical	-0.078	0.819
		MAX AP	0.014	0.968
		RMS ML	0.277	0.410
		RMS Vertical	-0.014	0.968
		RMS AP	0.014	0.968
		CEA	-0.230	0.495
	Eyes Closed	MAX ML	-0.051	0.882
		MAX Vertical	-0.332	0.319
		MAX AP	0.023	0.946
		RMS ML	0.277	0.410
		RMS Vertical	-0.014	0.968
		RMS AP	0.014	0.968
		CEA	-0.364	0.271
	Functional Reach	MAX ML	-0.191	0.596
		MAX Vertical	0.157	0.645
		MAX AP	-0.189	0.578
		RMS ML	-0.051	0.882
		RMS Vertical	0.327	0.326
		RMS AP	-0.203	0.550
		CEA	-0.300	0.371
	Functional Stability Boundary	MAX ML	-0.387	0.239
		MAX Vertical	0.032	0.925
		MAX AP	0.240	0.478
		RMS ML	0.327	0.326
		RMS Vertical	0.069	0.840
		RMS AP	0.018	0.957
		CEA	0.456	0.158
**Tee-shirt Test**	Eyes Open	MAX ML	-0.191	0.574
		MAX Vertical	0.191	0.574
		MAX AP	-0.336	0.312
		RMS ML	0.155	0.650
		RMS Vertical	0.336	0.312
		RMS AP	-0.336	0.312
		CEA	0.009	0.979
	Eyes Closed	MAX ML	0.391	0.235
		MAX Vertical	0.245	0.467
		MAX AP	-0.400	0.223
		RMS ML	0.155	0.650
		RMS Vertical	0.336	0.312
		RMS AP	-0.336	0.312
		CEA	0.182	0.593
	Functional Reach	MAX ML	0.358	0.310
		MAX Vertical	0.282	0.401
		MAX AP	0.164	0.631
		RMS ML	0.618	0.043*
		RMS Vertical	-0.127	0.709
		RMS AP	-0.091	0.790
		CEA	0.518	0.102
	Functional Stability Boundary	MAX ML	0.427	0.190
		MAX Vertical	0.118	0.729
		MAX AP	-0.236	0.484
		RMS ML	0.236	0.484
		RMS Vertical	0.027	0.937
		RMS AP	-0.318	0.340
		CEA	-0.200	0.555
**Forward Reach**	Eyes Open	MAX ML	0.533	0.091
		MAX Vertical	-0.592	0.055
		MAX AP	0.460	0.154
		RMS ML	0.615	0.044*
		RMS Vertical	-0.460	0.154
		RMS AP	0.460	0.154
		CEA	-0.214	0.527
	Eyes Closed	MAX ML	0.583	0.06
		MAX Vertical	-0.720	0.013*
		MAX AP	0.469	0.145
		RMS ML	0.615	0.044*
		RMS Vertical	-0.460	0.154
		RMS AP	0.460	0.154
		CEA	-0.560	0.073
	Functional Reach	MAX ML	-0.249	0.487
		MAX Vertical	-0.246	0.466
		MAX AP	0.560	0.073
		RMS ML	0.551	0.079
		RMS Vertical	-0.424	0.194
		RMS AP	0.428	0.189
		CEA	0.018	0.958
	Functional Stability Boundary	MAX ML	-0.246	0.466
		MAX Vertical	-0.574	0.065
		MAX AP	0.542	0.085
		RMS ML	0.633	0.036*
		RMS Vertical	-0.478	0.137
		RMS AP	0.401	0.222
		CEA	0.333	0.318
**Lateral Reach**	Eyes Open	MAX ML	0.780	<0.01**
		MAX Vertical	-0.413	0.207
		MAX AP	0.376	0.254
		RMS ML	0.706	0.015*
		RMS Vertical	-0.376	0.254
		RMS AP	0.376	0.254
		CEA	-0.174	0.608
	Eyes Closed	MAX ML	0.459	0.156
		MAX Vertical	-0.376	0.254
		MAX AP	0.376	0.254
		RMS ML	0.706	0.015*
		RMS Vertical	-0.376	0.254
		RMS AP	0.376	0.254
		CEA	-0.147	0.667
	Functional Reach	MAX ML	-0.255	0.476
		MAX Vertical	-0.624	0.040*
		MAX AP	0.183	0.589
		RMS ML	0.569	0.068
		RMS Vertical	-0.468	0.147
		RMS AP	0.330	0.321
		CEA	0.110	0.747
	Functional Stability Boundary	MAX ML	-0.220	0.515
		MAX Vertical	-0.211	0.533
		MAX AP	0.477	0.138
		RMS ML	0.826	<0.01**
		RMS Vertical	-0.651	0.030*
		RMS AP	0.385	0.242
		CEA	0.459	0.156
